# The renin-angiontensin system and the development of new antiglaucoma medications

**DOI:** 10.5935/0004-2749.20220095

**Published:** 2025-08-21

**Authors:** Aline Ruilowa de Pinho Coelho, Rodrigo da Silva Santos, Angela Adamski da Silva Reis

**Affiliations:** 1 Postgraduate Program in Health Science, Faculty of Medicine, Universidade Federal de Goiás, Goiânia, GO, Brazil; 2 Laboratory of Molecular Pathology, Department of Biochemistry and Molecular Biology, Institute of Biological Sciences, Universidade Federal de Goiás, Goiânia, GO, Brazil

Dear editor;

We have read the article entitled “Aqueous humor renin, angiotensin I, and angiotensin II activity in primary open-angle glaucoma”^(1)^ published in this esteemed journal. We would like to commend the authors because this is a well-written article with an extremely relevant theme. Additionally, we would like to raise few points regarding this study.

The selection of study participants could have followed specific exclusion criteria. The use of oral beta-blockers is known to weaken the response of intraocular pressure reduction when used concomitantly with beta-blocker eye drops for the treatment of glaucoma^(2)^. In addition, the use of beta-blocker eye drops in most participants with primary open-angle glaucoma may have influenced the final result of the concentration of renin being lower in the aqueous humor of participants with cataract and glaucoma than in the control group (i.e., patients with cataract only). The results presented by the authors contradict the current literature. Thus, for the intervention, it is best to withdraw treatment with beta-blocker eye drops for 15 days before collecting the aqueous humor. In cases where it is not possible to completely withdraw the medication, the beta-blocker eye drops can be substituted for another class of antiglaucomatous eye drops which does not directly interfere with renin activity.

Other important aspects that were not considered in the article are the participants’ stage of glaucoma and the number of topical medications for glaucoma that each participant was using at the time of the research. The ethnicities of the study participants were also not taken into account. This is important because black patients, for example, respond less to angiotensin-converting enzyme (ACE) inhibitors than white hypertensive patients. Furthermore, antihypertensive medications have similar dose-related effects among both black and white patients, but these are achieved at higher doses in the former^(3)^. Therefore, the doses of antihypertensive medications should also have been considered.

Angiotensin II has been implicated in retinal vascular diseases, such as retinopathy of prematurity and diabetic retinopathy, and recent studies on its relationship with neovascularization and glaucoma have emerged^(4)^. The reduction in angiotensin II production promoted by ACE inhibitors achieves a similar effect to beta-blockers, which reduce the activity of plasma renin^(5)^. It is up to us researchers to determine if the renin-angiotensin system is indeed involved in the pathogenesis of glaucoma, and, if possible, eventually develop new antiglaucoma medications with ACE inhibitors.
