# Supplementary material for: The renin-angiontensin system and the development of new antiglaucoma medications
Source: Arq Bras Oftalmol. 2025 Aug 21;85(2):211–3. doi: 10.5935/0004-2749.20220095 (PMC11826568; doi:10.5935/0004-2749.20220095)
Supplement: Related texts

# Reply: Response: Comments on The renin-angiotensin system and the development of new antiglaucoma medications

**Authors:** Valeria Batista Boreck Seki, Jayter Silva Paula

We would like to thank Aline R. P. Coelho and colleagues^(1)^ for their comments on our recent article, which evaluated the aqueous humor (AH) renin, angiotensin I, and angiotensin II activities in primary open-angle glaucoma (POAG). Our study has presented innovative findings displaying a remarkable reduction in AH renin activity among patients with POAG under timolol maleate topical treatment^(2)^. The comments of the authors raise a relevant point; significantly lower AH renin activity observed in patients with cataract and POAG might indeed be contradictory (a point also made in our original manuscript). Since high levels of renin may be implicated in modulating the intraocular pressure in glaucoma^(3)^, most patients with POAG showed lower renin activity than controls because they were using timolol maleate eye drops. Beta-blockers and angiotensin- converting enzyme (ACE) inhibitors may reduce the activity of plasma renin. Nonetheless, both the magnitude of this reduction and its cascade interactions are not entirely understood in the eye.

We agree with Coelho and colleagues that a washout period of beta-blocker eye drops would be ideal, as we have already mentioned in our discussion. However, a washout protocol should be clinically avoided since all included patients presented with severe POAG. They were under the maximum tolerated medication (hindering a drug switch) and even indicated for filtering surgery.

Although some of the local renin-angiotensin system (RAS) factors depend on interactions with circulatory RAS to fully operate, in some tissues, RAS may do it independently^(4)^. We agree that more information on race and oral medical treatment, including ACE inhibitors, is desired. However, the evidence of differential effects of antihypertensive medications in black patients as well as the local effect of systemic antihypertensives have not been completely studied in the eye. Furthermore, as presented in Table 1 of the manuscript, only a few patients across both groups were under systemic ACE inhibitors, and significantly fewer patients with POAG were treated with systemic beta-blockers.

Few studies have shown conditions that decrease AH renin activity. Neither a local increased consumption of renin nor its preferential bond switch to the prorenin receptor and consequent activation of this alternative RAS pathway have been studied in the eyes of patients with glaucoma. Since timolol maleate eye drops may reduce AH renin activity, the roles of renin and other RAS factors should be thoroughly investigated in various forms of the disease, including the study of different ocular target tissues, such as the trabecular meshwork. Further studies will help us fully understand the relationship between systemic and ocular RAS factors in glaucoma.

## References

[1] Coelho ARPC, Santos RS, Reis AAS (2021). The renin-angiontensin system and the development of new
antiglaucoma medications. Arq Bras Oftalmol..

[2] Seki VBB, Souza GR, Messias A, Casarini DE, Paula JS (2020). Aqueous humor renin, angiotensin I, and angiotensin II
activity in primary open-angle glaucoma. Arq Bras Oftalmol..

[3] Wang RF, Podos SM, Serle JB, Baltatu OC (2012). Effect of SPP 635, a renin inhibitor, on intraocular
pressure in glaucomatous monkey eyes. Exp Eye Res..

[4] Holappa M, Vapaatalo H, Vaajanen A (2017). Many Faces of Renin-angiotensin System - Focus on
Eye. Open Ophthalmol J..
